# Revolutionizing Hypertension Management in Type 2 Diabetes: The Promise of Digital Twin Technology

**DOI:** 10.1016/j.jacadv.2024.101173

**Published:** 2024-08-14

**Authors:** Anjan Tibrewala, Dipti Itchhaporia

**Affiliations:** a Division of Cardiology, Northwestern University, Chicago, Illinois; b Division of Cardiology, Hoag Hospital, University of California, Irvine, California

**Keywords:** digital health, digital twin, hypertension

**Figure:**
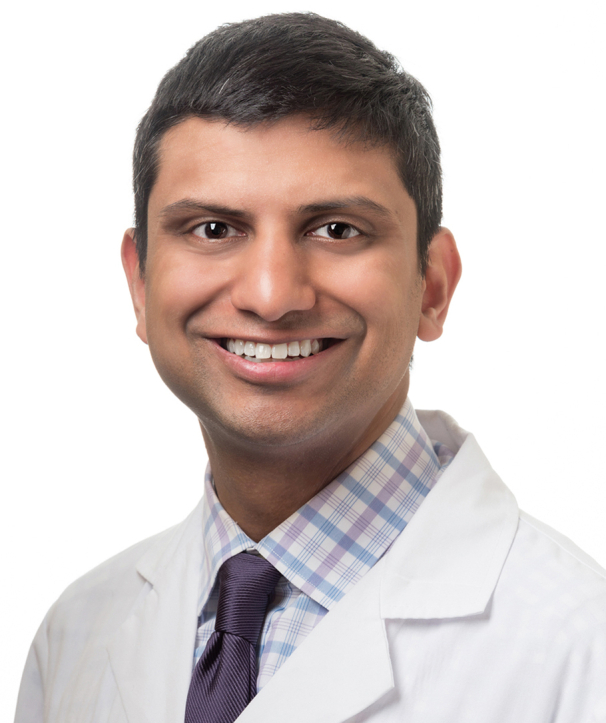

Digital health transformation is positioned to impact health care for patients and clinicians, aiming to optimize efficiency, improve outcomes, and reduce costs.^1^ In the realm of chronic disease management, the integration of advanced technologies has heralded a transformative approach to patient care. One such innovation is the Digital Twin (DT), a digital representation of an individual patient that periodically integrates and analyzes clinical data using mechanistic and statistical models. The goals of the DT technology are to improve diagnostics, inform prognosis, and guide treatments.^2^^,^^3^ DT technology is supported by various technologies, including artificial intelligence (AI), machine learning, and Internet of Things.^4^ Furthermore, DT technology provides personalized and precise lifestyle interventions, marking a significant departure from conventional methods.

In this context, hypertension (HTN) and type 2 diabetes mellitus (T2D) are globally prevalent conditions that have generated significant enthusiasm for digital health solutions. When concurrent, HTN and T2D significantly increase the risk of adverse cardiac events including incident coronary artery disease, stroke, and heart failure as well as microvascular complications.^5^ Thus, effective blood pressure (BP) control is essential for reducing mortality and morbidity in patients with T2D.^6^ Unfortunately, this goal is often unmet in clinical practice.

In this issue of *JACC: Advances*, Shamanna et al^7^ evaluated a DT intervention at large medical centers in India to improve BP control, reduce the use of anti-HTN medication, increase remission of HTN, and reduce microalbuminuria in patients with T2D within 1 year of follow-up. This study is a secondary analysis of a previously reported study that demonstrated the efficacy of DT in reducing hyperglycemia and metabolic-associated liver dysfunction in patients with T2D.^8^

In this study, the DT intervention consisted of digital sensors, including continuous glucose monitors, sensor watches, smart scales, and BP meters. These data were combined with clinical history, laboratory profiles, and food logs to form the DT, which then made tailored recommendations to improve medication adherence, nutrition, activity, and sleep. The patients were contacted via digital nudges (ie, text messages) and/or by a health coach. Physicians adjusted anti-HTN medications as necessary. In the standard care (SC) group, patients were managed according to standard HTN and T2D protocols. Home BP was only measured once a week and patients met with their physicians every 3 months for medication changes.^7^

Patients in the DT group experienced significantly greater reductions in both systolic and diastolic BP compared to the SC group. Among the 44 patients in the DT group initially on anti-HTN medications, 30 (68%) were able to stop taking anti-HTN agents as lifestyle changes were implemented, whereas none of the 15 patients in the SC group were able to stop taking such medications. Furthermore, patients in the DT group had significant reductions in microalbuminuria, while those in the SC group did not show any improvement. Thus, in this cohort of patients with HTN and T2D in India, a multifaceted DT intervention achieved significant improvements in several outcomes related to HTN compared to standard of care.

Prior studies have evaluated various digital interventions for HTN management, including reminders for medication adherence, apps for BP tracking, systems to support lifestyle modifications, education support, and platforms for provider-guided medication adjustments. These studies have had mixed results, often showing modest clinical benefits, especially when compared to the effects observed in the current trial.^9^^,^^10^ This raises the question: why was this DT intervention effective? The most unique aspect might be the AI-guided platform that integrated several data points to inform real-time feedback to patients in multiple health domains. Another major advantage of the AI-guided platform is that it can learn over time which particular interventions are most important for an individual patient—allowing for more personalized medicine.^2^^,^^11^ Patients in the DT group had enhanced interactions with the health care team including digital nudges, virtual interactions with a health coach, and more frequent medication adjustments, which could have also contributed to the observed improvements.

However, the study's limitations should be acknowledged. The small sample size (319 participants) and short follow-up period (1 year) constrain the generalizability of the results. These findings call for larger long-term studies to validate these findings. Additionally, the initial 6:1 randomization ratio between DT and SC groups, later adjusted to 2:1, might introduce allocation bias and affect the study's balance and validity. The study's ethnocultural context in India might affect its applicability to other populations and suggests the need for further research across diverse demographics. The other question is whether selection bias is impacting the DT cohort in which 68% of participants were able to stop taking anti-HTN agents. Is this due to this cohort have milder HTN than encountered in clinical practice?

Understanding the underlying mechanisms through which DT technology achieves these outcomes will be crucial for refining and optimizing its application. Objective data on the individual components of the overall intervention are essential. For example, data on the frequency of sensor measurements, app engagement, digital literacy, frequency of digital nudges, number of health coach interactions, and frequency of medication titrations would be informative for interpreting the current study. Additionally, the control group could have been better balanced against the intervention group. For example, the SC group could have had more access to a health coach or more physician-guided medication adjustments.

Another point to highlight is that implementing DT interventions can be resource intensive. These interventions require equipment (eg, sensors, smartphones), software technology, infrastructure to interface with health systems, and personnel. Moreover, they place demands on patients, potentially affecting how people cook, eat, sleep, move, and take medications. Therefore, it is crucial to focus efforts to optimize efficiency, particularly to allow equitable access to the intervention in settings with less resources.

Overall, Shamanna et al demonstrated that a DT intervention significantly improved the management of HTN for patients with T2D in India compared to standard of care.^7^ The significant reduction in BP, medication dependency, and microalbuminuria among the DT group participants highlights the potential of technology-driven interventions to enhance patient outcomes and reduce the burden of chronic disease management. This study on DT technology, by harnessing the power of AI and continuous monitoring, offers a personalized, proactive, and effective approach to chronic disease management. This represents a promising advancement, and further research and development will be key to unlocking its full potential.

## Funding Support and Author Disclosures

The authors have reported that they have no relationships relevant to the contents of this paper to disclose.
